# Fascin-1 is Highly Expressed Specifically in Microglia After Spinal Cord Injury and Regulates Microglial Migration

**DOI:** 10.3389/fphar.2021.729524

**Published:** 2021-09-27

**Authors:** Shuisheng Yu, Li Cheng, Dasheng Tian, Ziyu Li, Fei Yao, Yang Luo, Yanchang Liu, Zhenyu Zhu, Meige Zheng, Juehua Jing

**Affiliations:** ^1^ Department of Orthopaedics, The Second Hospital of Anhui Medical University, Hefei, China; ^2^ School of Pharmacy, Anhui Medical University, Hefei, China; ^3^ Department of Anatomy, Zhongshan School of Medicine, Research Center for Neurobiology, Sun Yat-Sen University, Guangzhou, China

**Keywords:** Fascin-1, microglia, polarization, migration, spinal cord injury

## Abstract

Recent research indicates that after spinal cord injury (SCI), microglia accumulate at the borders of lesions between astrocytic and fibrotic scars and perform inflammation-limiting and neuroprotective functions, however, the mechanism of microglial migration remains unclear. Fascin-1 is a key actin-bundling protein that regulates cell migration, invasion and adhesion, but its role during SCI has not been reported. Here, we found that at 7–14 days after SCI in mice, Fascin-1 is significantly upregulated, mainly distributed around the lesion, and specifically expressed in CX3CR1-positive microglia. However, Fascin-1 is not expressed in GFAP-positive astrocytes, NeuN-positive neurons, NG2-positive cells, PDGFRβ-positive cells, or blood-derived Mac2-positive macrophages infiltrating into the lesion core. The expression of Fascin-1 is correspondingly decreased after microglia are specifically depleted in the injured spinal cord by the colony-stimulating factor 1 receptor (CSF1R) inhibitor PLX5622. The upregulation of Fascin-1 expression is observed when microglia are activated by myelin debris *in vitro*, and microglial migration is prominently increased. The inhibition of Fascin-1 expression using small interfering RNA (siRNA) markedly suppresses the migration of microglia, but this effect can be reversed by treatment with myelin. The M1/M2-like polarization of microglia does not affect the expression of Fascin-1. Together, our results suggest that Fascin-1 is highly expressed specifically in microglia after SCI and can play an important role in the migration of microglia and the formation of microglial scars. Hence, the elucidation of this mechanism will provide novel therapeutic targets for the treatment of SCI.

## Introduction

Spinal cord injury (SCI) induces a complex heterogeneous inflammatory response largely mediated by resident microglia and infiltrating monocyte-derived macrophages (Davies and Miron, 2018; Milich et al., 2019). It has been difficult to distinguish between activated microglia and infiltrating macrophages in the injured spinal cord, since they share many similar markers and phenotypes (David and Kroner, 2011). Recent advances in conditional gene targeting analysis have allowed the identities and functions of these cell populations to become increasingly clear (Milich et al., 2019; Zhou et al., 2020). It has been shown that after SCI, infiltrating macrophages expressing high Mac2 levels but low CX3CR1 levels accumulate in the lesion core, which are overloaded with myelin debris, and trigger a sustained inflammatory reaction (Zhou et al., 2014; Wang et al., 2015). In contrast, resident microglia expressing high CX3CR1 levels accumulate around the lesion core and form a border, which is named the “microglial scar”, to seal the lesion and block the spread of damage (Bellver-Landete et al., 2019). However, the mechanism of microglial scar formation is far from clear.

Fascin-1, an actin-bundling protein, plays a key role in the assembly and stability of cell protrusions and other actin-based structures that aid in cell motility, migration and invasion (Tan et al., 2013). A multitude of studies have shown that Fascin-1 supports the migratory and metastatic capacities of carcinomas (Hashimoto et al., 2011; Ma et al., 2013; Gao et al., 2019). In addition, it has recently been reported that Fascin-1 is upregulated predominantly in the microglia in the dorsal horn of the spinal cord in a rat chronic constriction injury of the sciatic nerve model of neuropathic pain and that Fascin-1 contributes to neuropathic pain by promoting inflammation (Wang et al., 2018). However, the cellular localization and function of Fascin-1 after SCI have not been reported.

In the present study, we found that Fascin-1 was highly expressed specifically in microglia that accumulated at the lesion border after SCI. Fascin-1 expression decreased accordingly after the specific depletion of microglia in the injured spinal cord, and this effect was accompanied by disorganized astrocytic and fibrotic scars and scattered macrophages in the injured spinal cord. Fascin-1 knockdown markedly suppressed microglial migration, but this effect could be reversed by myelin treatment. Our results suggest that Fascin-1 may play a key role in microglial migration and microglial scar formation in SCI.

## Materials and Methods

### Animals

All the animal procedures were performed in accordance with the guidelines of the Institutional Animal Care and Use Committee of Anhui Medical University (No. LLSC20160052). C57BL/6J female mice at 6–8 weeks of age were purchased from the Experimental Animal Center of Anhui Medical University. The mice were housed in a temperature- and humidity-controlled room with a 12:12-light/dark cycle and allowed free access to food and water.

### Spinal Cord Injury Model

All the surgical procedures were performed under pentobarbital anesthesia. The skin around the lesion core was shaved and disinfected using iodophor. Subsequently, the T10 spinal cord was exposed *via* a dorsal laminectomy, which was located spinous process of mice, cut open the back skin about 2 cm, separate soft tissue to posterior lamina and open with rongeur to expose the spinal cord, and moderately severe crush SCIs were made using No. 5 Dumont forceps (Fine Science Tools, 11,252–20, Heidelberg, Germany) ground down to a tip with a width of 0.5 mm by compressing the cord laterally from both sides for 5 s (Wanner et al., 2013). Then, twitching of the hind limbs and movement of the tail were observed, which indicated that the SCI model was successfully established. Finally, the wound was sutured with 3–0 silk threads. The mice with SCI were examined daily to monitor their recovery, and their bladders were expressed manually three times a day until the return of reflexive bladder control. The sham group were subjected to laminectomy alone. The mice were sacrificed at 3, 7 and 14 days after SCI.

### Microglia Depletion

To eliminate microglia, mice were administered PLX5622 (MedChemExpress, HY-114153) at 130 mg/kg by oral gavage once a day for 17 consecutive days. PLX5622 was diluted in 5% DMSO, 40% polyethylene glycol 300, 5% polysorbate 80, and 50% saline, according to the manufacturer’s instructions. The total volume of the liquid is 200 μl An equal volume of vehicle was used as the control. SCI was established on the third day after gavage.

### Preparation of Myelin Debris

Myelin debris was isolated as previously described (Rolfe et al., 2017). Briefly, 6- to 8-week-old mice were euthanized, and their brain tissue was harvested and homogenized in ice-cold 0.32 M sucrose. Myelin debris was isolated from the brain tissue by sucrose density gradient centrifugation. The endotoxin concentration of the myelin debris was below the limit of detection of the Limulus Amebocyte Lysate assay (Lonza, Switzerland). Myelin debris was added to cells at a final concentration of 1 mg/ml in all the experiments.

### Cell Culture and Transfection

BV-2 is a commonly used microglial cell line that has been widely used *in vitro* experiments to explore inflammatory responses in many studies (Riedl et al., 2009; Basso et al., 2017; Yoshizaki et al., 2021). The BV-2 microglial cell line was obtained from the American Type Culture Collection (CRL-3265, ATCC, Manassas, VA, United States) and cultured in Dulbecco’s modified Eagle’s medium (DMEM, HyClone, SH30021) supplemented with 10% fetal bovine serum (FBS, Gibco, 10,270,106), 100 U/ml penicillin and 100 g/ml streptomycin (Gibco, Grand Island, NY, United States). The cells were incubated in a humidified chamber at 37°C in a 95O_2_ and 5% CO_2_ atmosphere. Small interfering RNA (siRNA) was transfected into these cells using jetPRIME (Polyplus Transfection, 114–15), according to the manufacturer’s instructions. siRNA targeting mouse Fascin-1 (siRNA: 5′- GAU​GCC​AAC​CGU​UCC​AGU​UTT -3′) and nonspecific control siRNA (NC) were purchased from GenePharma (Shanghai, China).

### Microglial Polarization

BV-2 cells were plated in poly-d-lysine (PDL, Sigma, P7280)-coated 6-well plates at a density of 1 × 10^6^ cells/ml and cultured overnight. After serum deprivation for 24 h, the BV-2 cells were polarized toward the M1-like phenotype by treatment with lipopolysaccharide (LPS; 100 ng/ml, Beyotime Biotechnology, ST1470, Shanghai, China) and IFNγ (20 ng/ml, Beyotime Biotechnology, P6137) or toward the M2-like phenotype by treatment with IL-4 (20 ng/ml, Beyotime Biotechnology, P5916), and the cells were cultured for 24 h (Kigerl et al., 2009; David and Kroner, 2011). The supernatant was taken as the conditioned medium (1200 rpm, 5 min) after 24 h culture in serum-free medium. The M1 and M2 polarization of BV2 cells was identified by subsequent Western blot analysis and immunofluorescence analysis.

### Tissue Processing

For Western blot analysis, the mice were anesthetized and transcardially perfused with 0.1 M phosphate-buffered saline (PBS) to remove the blood. Spinal cord tissue of 5 mm centered at the lesion core were harvested. For histological analysis, once the blood was removed, the mice were transcardially perfused with 4% paraformaldehyde (PFA), and a 5 mm portion of the spinal cord encompassing the injury epicenter was extracted and embedded in paraffin. Then, the tissue was sagittally sectioned at thicknesses of 6 μm on a microtome (Leica RM2235). Every 10th section was collected and mounted onto a series of slides.

### Immunofluorescence Analysis

Immunohistochemistry. Six-micrometer, paraffin-embedded spinal cord sections were dried, dewaxed, hydrated and subjected to antigen repair. Next, 10% donkey serum albumin (DSA, Solarbio, SL050) containing 0.3% Triton X-100 (Solarbio, T8200) was added and incubated at room temperature for 1 h. Then, primary antibodies were added and incubated at 4°C overnight. The primary antibodies used were as follows: mouse anti-Fascin-1 (1:50, Santa Cruz, sc-21743), rabbit anti-Fascin-1 (1:100, Abcam, ab126772), goat anti-Iba1 (1:200, Novus Biologicals, NB100-1028), rabbit anti-CX3CR1 (1:500, Abcam, ab8021), rabbit anti-Tmem119 (1:100, Synaptic system, 400002), rabbit anti-GFAP (1:100, Proteintech, 16825-1-AP), mouse anti-GFAP (1:100, Proteintech, 60190-1-Ig), goat anti-PDGFRβ (1:40, R&D Systems, AF1042-SP), rabbit anti-PDGFRβ (1:200, Abcam, ab32570), mouse anti-Mac2 (also known as galectin-3, 1:100, GB12246, Servicebio), rabbit anti-NG2 (1:100, Proteintech, 55027-1-AP), rabbit anti-iNOS (1:100, Affinity, AF0199), rabbit anti-CD206 (1:100, ab64693, Abcam) and rabbit anti-NeuN (1:500, Abcam, ab177487). The secondary antibodies were diluted in 1% donkey serum in PBS and incubated for 1 h at room temperature. The following secondary antibodies were used: Alexa Fluor 488 and Alexa Fluor 594 (1:500, Thermo Fisher Scientific, A-21206, A-21202, A-21203, A-21207, A-11058). The nuclei were stained using 4’,6-diamidino-2-phenylindole (DAPI) (1 μg/ml, Thermo Fisher Scientific). The fluorescence signals were obtained using an Axio Scope A1 microscope (Zeiss, Germany). ImageJ software (National Institutes of Health, Bethesda, MD, United States) was used for quantitative analysis.

Immunocytochemistry. Cells were fixed with 4% PFA for 10–15 min, permeabilized in 0.5% Triton X-100 in PBS for 10 min and blocked with 5% donkey serum in PBS for 30 min at 20–25°C. The primary antibodies (as listed above) were diluted in 1% donkey serum in PBS and incubated overnight at 4°C. The secondary antibodies (as listed above) were diluted in 1% donkey serum in PBS and incubated for 1 h at room temperature. Images were acquired as described above.

### Imaging Analysis and Quantification

Every 10th 6 μm-thick, paraffin-embedded section was quantified, resulting in five analyzed slides per animal that included the entire injured spinal cord. The total numbers of Fascin-1^+^, CX3CR1^+^, and Fascin-1^+^CX3CR1^+^ double-positive cells in each of the sagittal sections of the spinal cord was counted under a ×20 objective lens with Zeiss ZEN imaging software (Zeiss, Germany).

### Western Blot Analysis

The injured spial cord tissue was homogenized in radioimmunoprecipitation assay (RIPA) buffer (Sigma, R0278) supplemented with protease inhibitors (Roche, 04693124001) and phosphatase inhibitors (Roche, 04906845001). The cells were washed with cold PBS, homogenized in RIPA buffer on ice for 30 min, and then centrifuged at 12,000 rpm at 4°C for 30 min. The protein extracts were quantified by using a Pierce BCA protein assay kit (Beyotime Biotechnology, P0010S). Aliquots of the protein samples containing equal protein concentrations were separated on 10% sodium dodecyl sulfate (SDS)-polyacrylamide gels and subsequently transferred to polyvinylidene (PVDF) membranes. The membranes were blocked with 5% nonfat milk in Tris-buffered saline with 0.5% Tween-20 (TBST) at room temperature for 1 h and then incubated with primary antibodies, including mouse anti-GAPDH (1:2000, Proteintech, 60004-1-Ig), rabbit anti-Fascin-1 (1:5000, Abcam, ab126772), rabbit anti-iNOS (1:3000, AF0199, Affinity) and rabbit anti-CD206 (1:1000, ab64693, Abcam), at 4°C overnight. After three washes with TBST, the membranes were incubated with horseradish peroxidase (HRP)-conjugated goat anti-mouse secondary antibodies (1:10,000, Sigma, A4416) and HRP-conjugated goat anti-rabbit secondary antibodies (1:10,000, Sigma, A0545) for 1 h at room temperature. The protein band signals were obtained using an ECL detection kit (ECL, Thermol Biotech Inc., United States) and Tanon 5,200 system (Tanon, Shanghai, China). ImageJ was used for quantification analysis. The intensity of the GAPDH bands was used for normalization.

### Scratch Assay

Cell migration was assessed by performing a scratch assay. Briefly, BV-2 cells were seeded into PDL-coated 6-well plates at a density of 2 × 10^5^ cells/well and incubated for 24 h. Then, BV2 cells were transfected with NC or siFascin-1 according to different design groups. After 24 h with transfection, BV2 cells were cultured with serum-free medium and were treated with or without myelin debris at a final concentration of 1 mg/ml for another 24 h. Then, BV2 cells were washed away with PBS for three times to remove non-ingested myelin debris. The cell layers were scratched using a 200-µl pipette tip to form a wound-like gap. The cells were then maintained in DMEM with 2% FBS, and images were captured at 0, 24 and 48 h after cell scratching. ImageJ was used to analyze the wound width.

### Transwell Assay

A 24-well plate containing a 3 μm chamber (Costar, 3415) was used to assess the migration abilities of the cells. BV-2 cells were transfected, treated with myelin debris and activated in a PDL-coated 6-well plate before inoculation into the chamber. Then, the cells were suspended in serum-free media and seeded into the upper chamber (5 × 10^4^ cells per chamber). The lower chamber contained complete medium. After incubating for 12 h, the nonmigrating cells on the inside of the membrane were carefully removed with a cotton swab, and the migrating cells on the outside of the membrane were fixed with 4% PFA at room temperature for 20 min and stained with 0.1% crystal violet for 15 min. The cells were observed under a microscope and counted. At least five random fields were photographed, and the cells in each field were counted.

### Statistical Analysis

All the experiments were independently performed with at least three replicates and quantified in a blinded manner. The data are presented as the mean ± standard error of the mean (SEM). The statistical analysis was carried out with SPSS version 19.0 (SPSS Inc., Chicago, IL, United States) software. *Student’s t* test was used to compare the difference between two groups. One-way analysis of variance followed by *Tukey’s post hoc* test was used to compare differences among multiple groups. Differences were considered statistically significant if *p* < 0.05.

## Results

### Fascin-1 Is Significantly Upregulated and Distributed Outside the Lesion Core After Spinal Cord Injury

**FIGURE 1:**
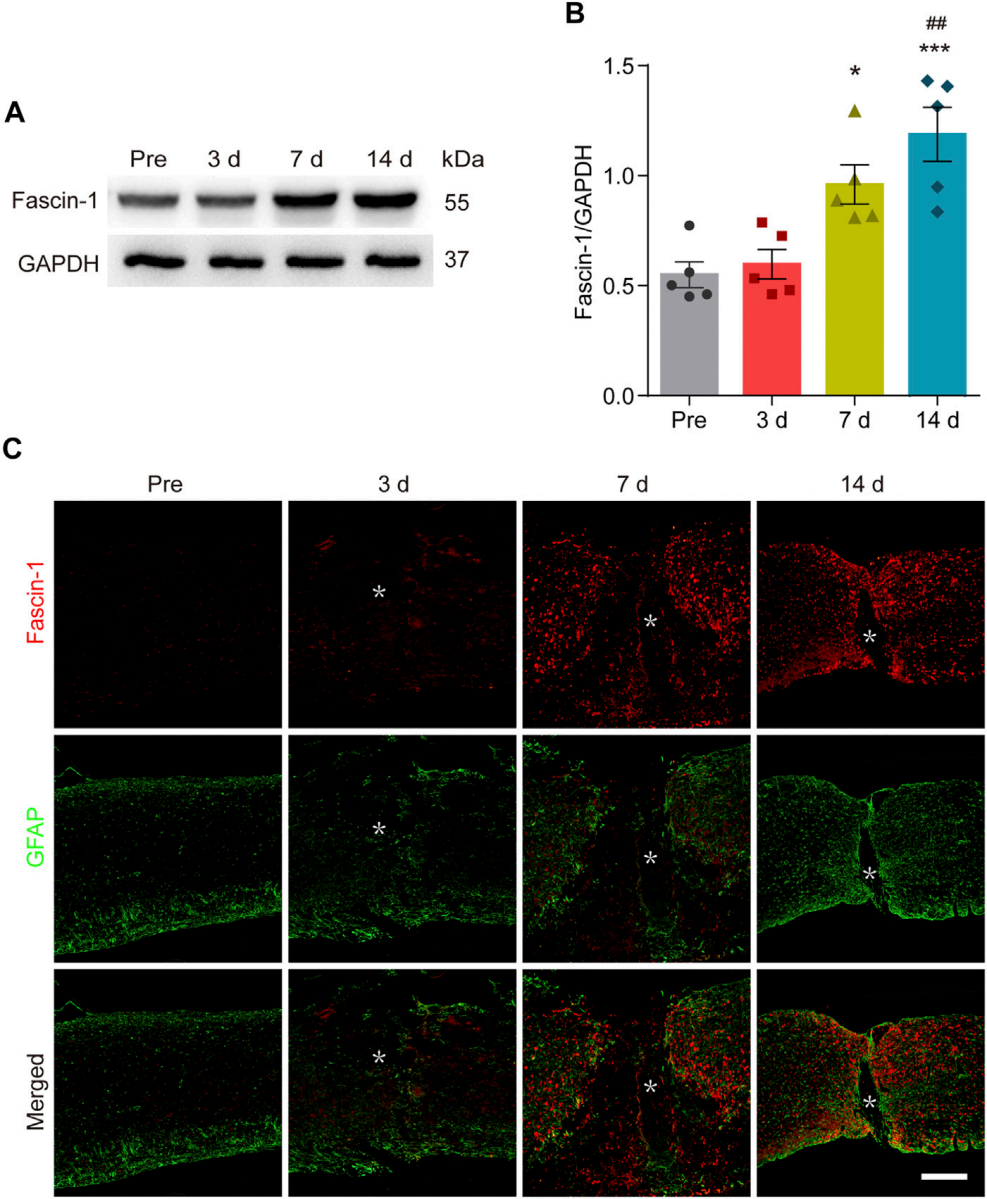
Fascin-1 is prominently expressed and accumulates outside the lesion core after SCI. **(A)** Western blot analysis shows significant upregulation of Fascin-1 at 7 and 14 days after injury compared to the control (pre-operation, Pre). **(B)** Quantitative analysis of Fascin-1 expression in **(A)**. The blots (*n* = 5 per group) were quantified by a densitometric method using ImageJ software. GAPDH was used as the loading control. The results are expressed as the mean ± SEM. ***p < 0.001 (14 days vs Pre); ^*^ *p* < 0.05 (7 days vs Pre); ##*p* < 0.01 (14 vs 3 days). **(C)** Double immunofluorescence labeling of spinal cord sagittal sections showing the spatiotemporal distribution of Fascin-1 (Red) and GFAP (Green) at Pre and 3, 7, and 14 days after injury. The asterisks indicate the lesion epicenter. Scale bar: 500 μm.

To determine the changes in Fascin-1 expression during SCI, Western blot was used to detect the relative expression levels of Fascin-1 before and 3–14 days after SCI. As shown in Figures 1A,B, Fascin-1 expression was significantly increased at 7 and 14 days after SCI compared with that before injury (^*^ *p* < 0.05 and ^***^ *p* < 0.001, respectively). To further analyze the distribution of Fascin-1, we carried out immunofluorescence detection of Fascin-1 and GFAP, which is used to label the astrocytic scar formed around the lesion core. The fluorescence signal of Fascin-1 was also obviously increased at 7 and 14 days after SCI and gradually accumulated outside the lesion core, intermingling but not colocalizing with GFAP^+^ astrocytic scars (Figure 1C). These results indicate that the expression of Fascin-1 is prominently upregulated and mainly distributed outside the lesion core, Fascin-1 expression has a spatiotemporal distribution pattern similar to that of microglia after SCI (Bellver-Landete et al., 2019).

### Fascin-1 Is Specifically Expressed in Activated Microglia After Spinal Cord Injury

**FIGURE 2:**
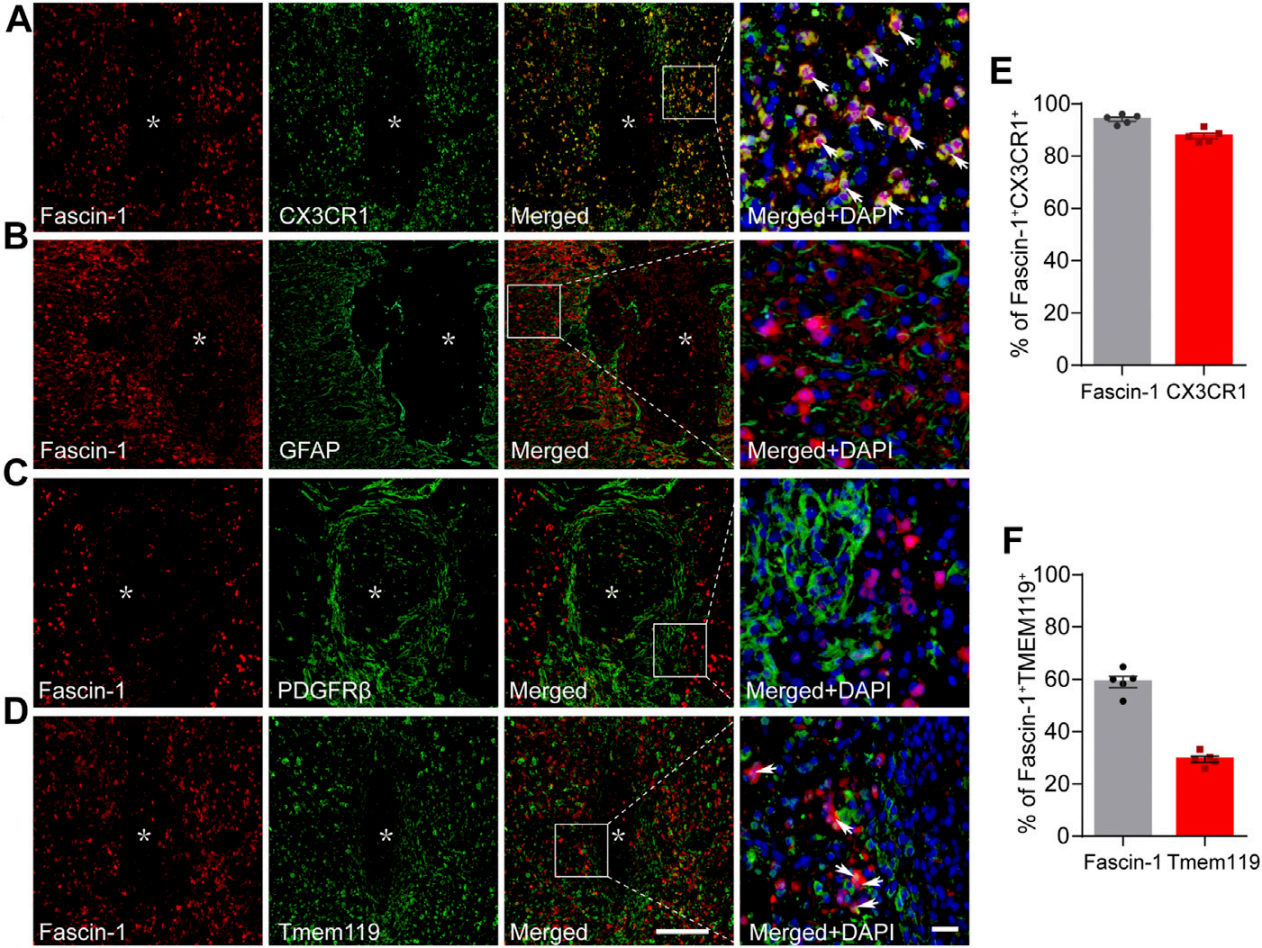
Fascin-1 is specifically expressed in CX3CR1^+^ activated microglia but not in GFAP^+^ astrocytes or PDGFRβ^+^ epicenter-located pericytes at 14 days after SCI. **(A)** Representative immunofluorescence images of Fascin-1 (Red) and CX3CR1 (Green). Colocalization of the proteins is shown in yellow. Nuclear staining (DAPI) is shown in blue, and white arrowheads indicate the colocalization observed with a ×40 objective lens. **(B)** Representative immunofluorescence images of Fascin-1 (Red), GFAP (Green) and DAPI (Blue) showing no apparent colocalization of staining. **(C)** Representative immunofluorescence images of Fascin-1 (Red), PDGFRβ (Green) and DAPI (Blue) showing no apparent colocalization of staining. The asterisks indicate the lesion epicenter. Scale bars: low magnification, 200 μm; higher magnification, 20 μm. **(D)** Representative immunofluorescence images of Fascin-1 (Red) and Tmem119 (Green). Arrowheads indicate the colocalization observed with a ×40 objective lens. **(E)** Percentage of Fascin-1^+^CX3CR1^+^ cells relative to the total number of Fasicn-1^+^ or CX3CR1^+^ cells in the injured spinal cord. **(F)** Percentage of Fascin-1^+^Tmem119^+^ cells relative to the total number of Fasicn-1^+^ or Tmem119^+^ cells in the injured spinal cord. The data are presented as the mean ± SEM (*n* = 5 independent experiments).

The cellular localization of Fascin-1 was then detected by immunofluorescence staining. We found that Fascin-1 was highly colocalized with CX3CR1-labeled microglia. The percentage of Fascin-1^+^CX3CR1^+^ costained cells relative to the total number of Fascin-1^+^ or CX3CR1^+^ cells in the injured spinal cord reached 94.06 ± 0.82% or 87.65 ± 1.08%, respectively (Figures 2A,E). These costained cells were mainly located around the SCI site (Figure 2A), which was consistent with previous studies (Bellver-Landete et al., 2019). Fascin-1 was not expressed in the GFAP^+^ astrocytes surrounding the lesion core or in the PDGFRβ^+^ pericytes accumulated in the epicenter (Figures 2B,C). We further examined the co-expression of Fascin-1 and Tmem119, a specific marker of homeostatic microglia (Bennett et al., 2016), the results showed that they were partially costained and confined outside of the lesion core (Figure 2D). The percentage of Fascin-1^+^ Tmem119^+^ costained cells relative to the total number of Fascin-1^+^ or Tmem119^+^ cells in the injured spinal cord reached 29.38 ± 1.19% or 59.0 ± 12.13%, respectively (Figure 2F). Considering the high co-staining of Fascin-1 and CX3CR1 (Figure 2A), these suggest that Fascin-1 may be significantly up-regulated in the activated microglia.

**FIGURE 3:**
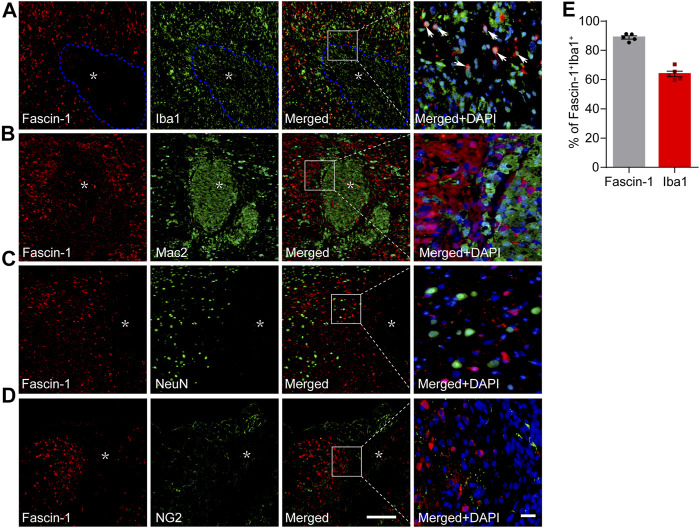
Fascin-1 is expressed partly in Iba1^+^ cells around the lesion but not in infiltrating Mac2^+^ macrophages, NeuN^+^ neurons or NG2^+^ cells in the injured spinal cord at 14 days. **(A)** Representative immunofluorescence images of Fascin-1 (Red) and Iba1 (Green). Arrowheads indicate the colocalization observed with a ×40 objective lens. The blue dotted lines delineate the boundary around the lesion core. **(B–D**) Representative immunofluorescence images of Fascin-1 (Red) staining with Mac2, NeuN, and NG2 (Green) staining, respectively, showing no apparent colocalization of staining. The asterisks indicate the lesion epicenter. Nuclear staining with DAPI is shown (Blue). Scale bars: low magnification, 200 μm; higher magnification, 20 μm. **(E)** Percentage of Fascin-1^+^Iba1^+^ cells relative to the total number of Fasicn-1^+^ or Iba1^+^ cells outside the blue dotted lines in the injured spinal cord. The data are presented as the mean ± SEM (*n* = 5 independent experiments).

Iba1 is a common marker of microglia, but during SCI, both resident microglia and infiltrating macrophages can be identified by Iba1 (Wang et al., 2015). Our results showed that costaining of Fascin-1 and Iba1 was mainly confined around the lesion core, which was consistent with the localization of microglia (Figure 3A). Since Iba1 can also mark infiltrating macrophages, we used the area stained by Fascin-1 to identify the lesion border and circled it with the blue dotted line. We counted the costained of Fascin-1 and Iba1 outside the blue dotted line. The percentage of Fascin-1^+^Iba1^+^ costained cells relative to the total number of Fascin-1^+^ or Iba1^+^ cells in the injured spinal cord reached 88.79 ± 1.09% or 63.81 ± 1.93%, respectively (Figure 3E). Moreover, Fascin-1 was not colocalized with Mac2^+^ macrophages located at the lesion core (Figure 3B). In addition, Fascin-1 was not expressed in NeuN^+^ neurons or NG2^+^ cells in the injured area (Figures 3C,D). Taken together, these results show that Fascin-1 is highly expressed specifically in activated microglia after SCI.

### Depletion of Microglia Correspondingly Reduces Fascin-1 Expression After Spinal Cord Injury

**FIGURE 4:**
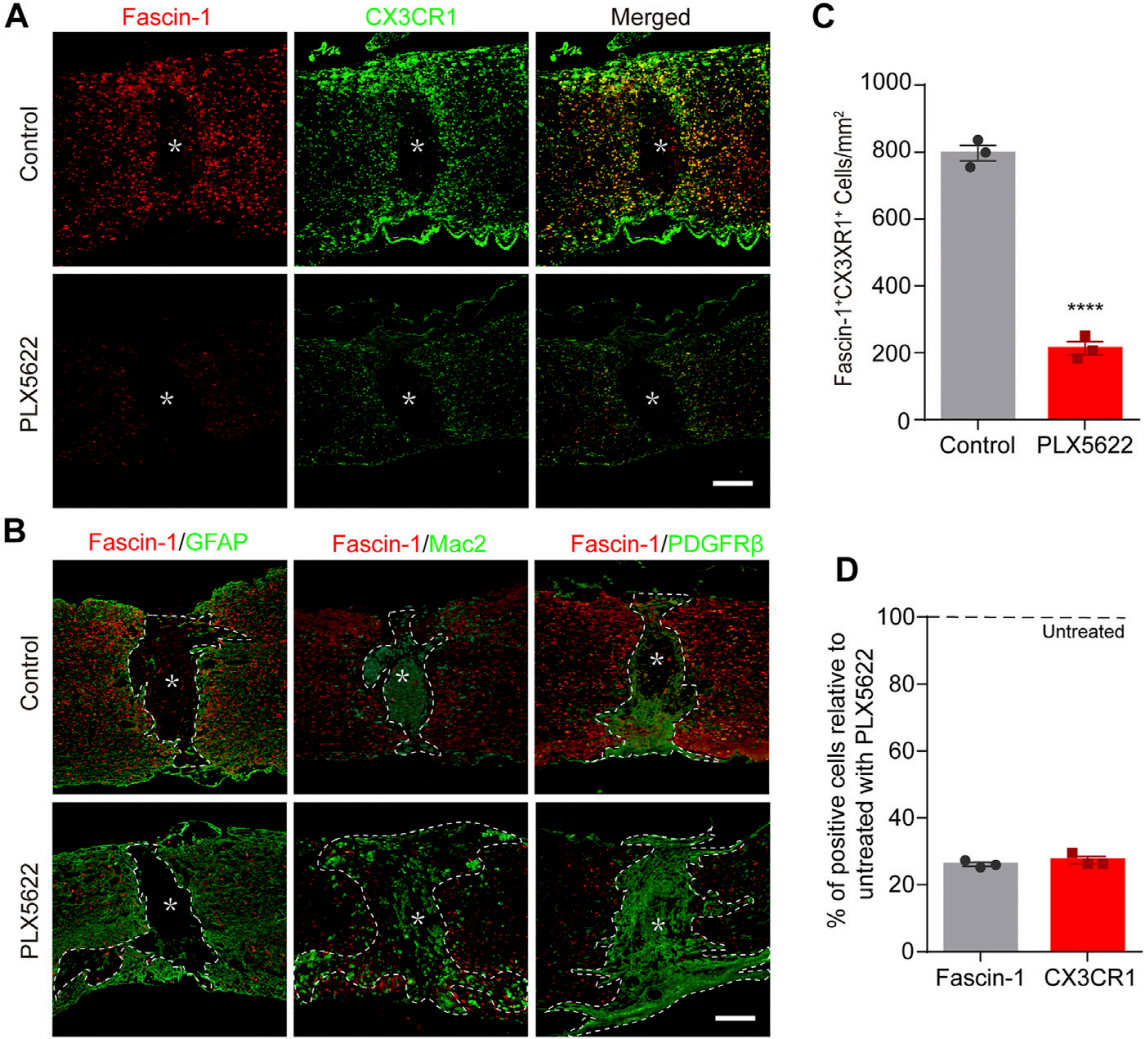
The elimination of microglia by PLX5622 treatment results in correspondingly reduced Fascin-1 expression, disorganized astrocytic and fibrotic scars, and scattered macrophages in the injured spinal cord at 14 days. **(A)** Representative fluorescence images of Fascin-1 (Red) and CX3CR1 (Green) immunostaining showing a consistent reduction in Fascin-1 expression with the elimination of microglia after treatment with PLX5622 compared to vehicle (control). The asterisks indicate the lesion epicenter. Scale bar: 500 μm. **(B)** Representative immunofluorescence images of Fascin-1 (Red) and GFAP (Green), Fascin-1 (Red) and Mac2 (Green), Fascin-1 (Red) and PDGFRβ (Green) at the lesion sites of mice treated with vehicle (control) or PLX5622. After the elimination of microglia using PLX5622, the compact GFAP^+^ astrocytic scars and PDGFRβ^+^ fibrotic scars are disrupted, with clusters of Mac2^+^ macrophages spreading outside of the lesion core. The asterisks indicate the lesion epicenter. The dotted lines delineate the boundary around the lesion core. Scale bar: 500 μm. **(C)** Quantification of the number of Fascin-1^+^ CX3CR1^+^ microglia in the control and PLX5622 groups (*n* = 3 per group). The results are expressed as the mean ± SEM. ****p < 0.0001. **(D)** Percentage of surviving Fascin-1^+^ or CX3CR1^+^ cells in the PLX5622 groups relative to that the untreated control groups (*n* = 3 per group).

To confirm that Fascin-1 is derived from microglia, PLX5622, a selective inhibitor of colony-stimulating factor 1 receptor (CSF1R) that crosses the blood–brain barrier (BBB) and eradicates nearly all the microglia in the central nervous system (CNS), was administered to mice (Huang et al., 2018). A solution containing PLX5622 was administered to mice by gavage starting 3 days prior to SCI and continuing until sacrifice. As shown in Figures 4A,C, the number of Fascin-1^+^CX3CR1^+^ microglia in the PLX5622 group was significantly decreased compared to that in the control group (****p < 0.0001). The expression of Fascin-1 decreased accordingly with the depletion of CX3CR1^+^ microglia. The percentages of Fascin-1^+^ cells and CX3CR1^+^ cells in the PLX5622 groups relative to those in the untreated control groups were 26.14 ± 0.61% and 27.38 ± 1.07%, respectively (Figure 4D). After the depletion of microglia, we observed that both GFAP^+^ astrocytic scars and PDGFRβ^+^ fibrotic scars became less compact and disorganized, and infiltrating Mac2^+^ macrophages were diffusely scattered outside of the lesion core (Figure 4B). Fascin-1 may be involved in microglial scars formation, which needs to be confirmed by specifically knocking out microglia-derived Fascin-1 instead of depleting microglia.

### High Expression of Fascin-1 can Promote Microglial Migration

**FIGURE 5:**
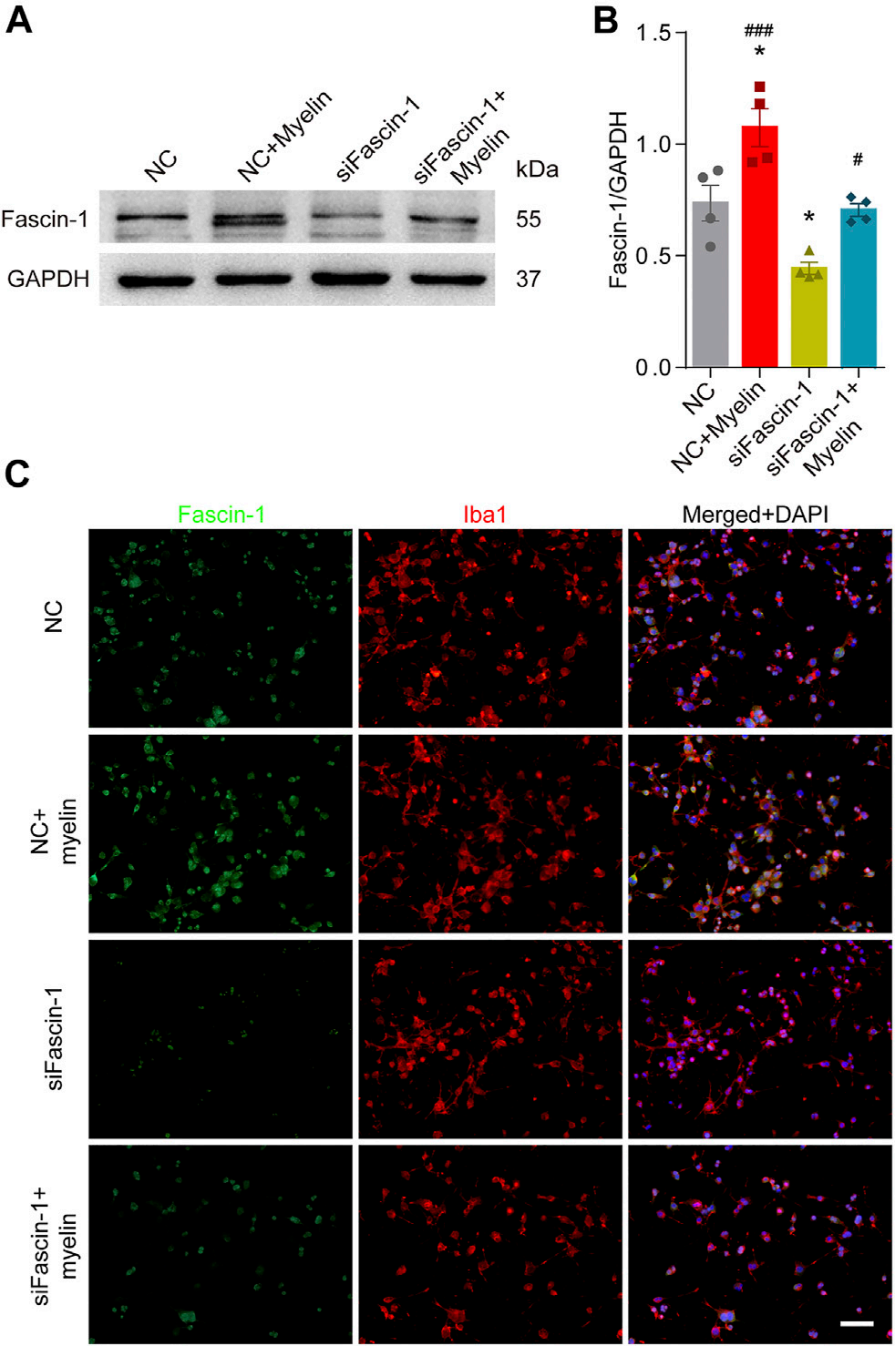
Inhibition of Fascin-1 expression using siRNA in BV-2 microglia can be partly rescued by treatment with myelin. **(A)** Western blot analysis shows that compared to the nonspecific control (NC), Fascin-1 siRNA (siFascin-1) could knockdown the expression of Fascin-1, while myelin treatment could upregulate the expression of Fascin-1. **(B)** Quantitative analysis of Fascin-1 expression in **(A)**. GAPDH was used as the loading control. The blots (*n* = 4 per group) were quantified as previously described. The results are expressed as the mean ± SEM. ^*^ *p* < 0.05 (NC + Myelin or siFascin-1 vs NC); ^#^ *p* < 0.05 (NC + Myelin vs siFascin-1), ^###^ *p* < 0.001 (siFascin-1+Myelin vs siFascin-1). **(C)** BV-2 cells were transfected with nonspecific control (NC) siRNA or siFascin-1 for 24 h and then treated with or without myelin for an additional 24 h in complete medium. Representative immunofluorescence images of Fascin-1 (Green) and Iba1 (Red) are shown. DAPI (Blue) was used to stain the nuclei. Scale bar: 100 μm.

**FIGURE 6:**
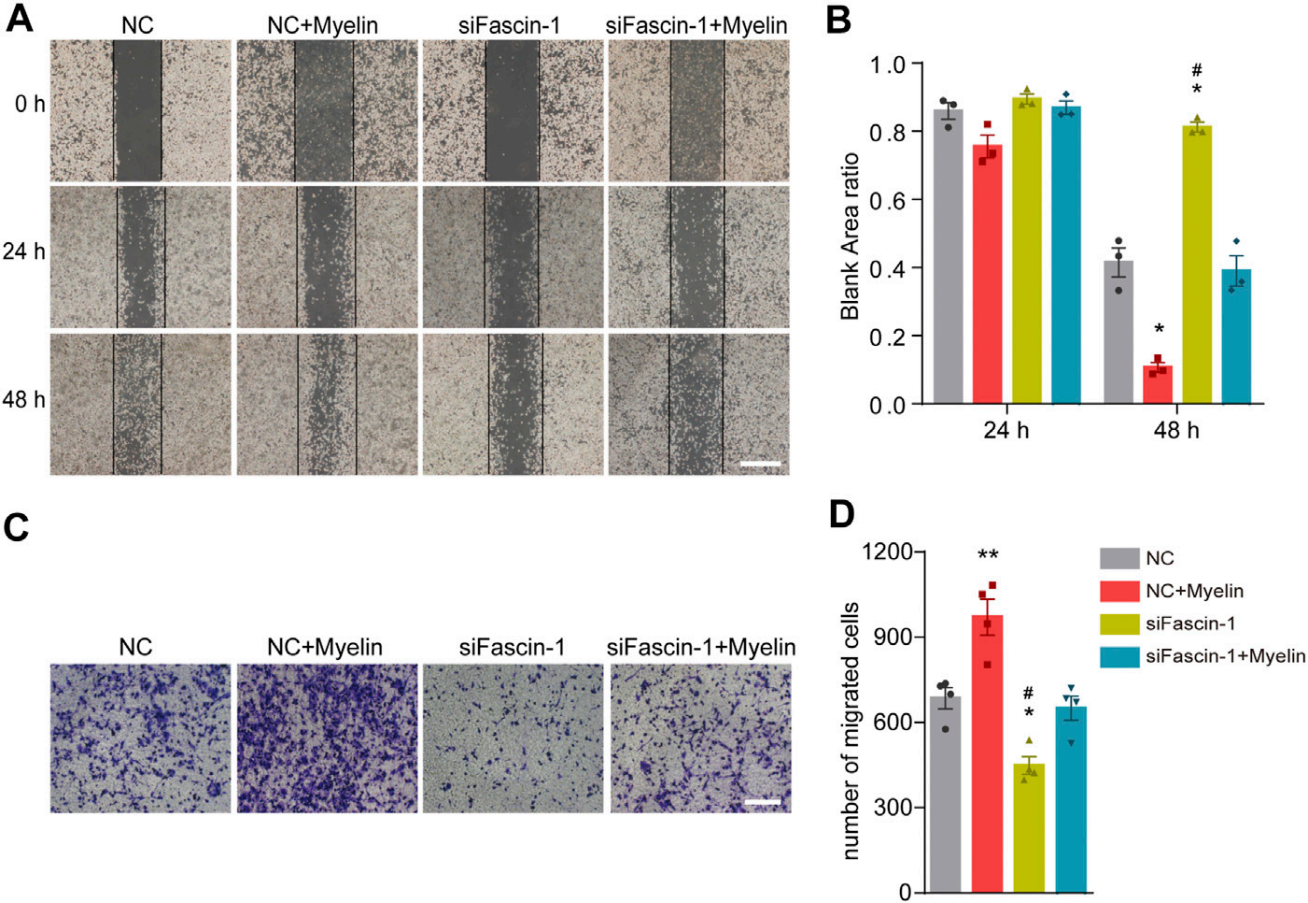
Fascin-1 promotes the migration of microglia *in vitro*. **(A)** For the scratch assay, cell migration was recorded at 0, 24 and 48 h after scratching (n = 3 per group). BV-2 cells were transfected with nonspecific control (NC) or Fascin-1 (siFascin-1) siRNA for 24 h and treated with or without myelin for an additional 24 h in complete medium. Scale bar: 2 mm. **(B)** Quantification of the ratio of the blank area in the scratch migration assay. The results are presented as the mean ± SEM of experiments conducted in triplicate. ^*^ *p* < 0.05 (NC + Myelin or siFascin-1 vs NC); ^#^ *p* < 0.05 (NC + Myelin vs siFascin-1). **(C)** Transwell assays were used to detect the migration of microglia. BV-2 cells were treated as described in **(A)**. After 48 h, the cells were resuspended in serum-free media and added to the upper chamber. Then, the cells were allowed to migrate for an additional 12 h and stained with crystal violet. Scale bar: 200 μm. **(D)** Quantitative analysis of the number of transmembrane cells in **(C)** (*n* = 4 per group). The results are presented as the mean ± SEM. **p < 0.01 (NC + Myelin vs NC), ^*^ *p* < 0.05 (siFascin-1 vs NC). ^#^ *p* < 0.05 (siFascin-1 vs siFascin-1+Myelin).

To assess the effect of Fascin-1 on the function of microglia, we cultured the BV-2 microglial cell line *in vitro*. Western blot (Figures 5A,B) and immunocytochemistry (Figure 5C) analyses revealed that compared to the negative controls, Fascin-1 siRNA (siFascin-1) could knockdown the expression of Fascin-1, while myelin treatment could upregulate the expression of Fascin-1 (^*^ *p* < 0.05). The migration ability of the microglia was reduced after Fascin-1 knockdown but enhanced by myelin addition, as observed by scratch and Transwell assays (^*^ *p* < 0.05, Figure 6). Furthermore, the inhibition of Fascin-1 expression and microglial migration using siFascin-1 could be reversed by treatment with myelin (Figures 5, 6). These results suggest that Fascin-1 is required for typical microglial migration.

### Polarization of Microglia Does not Affect the Expression of Fascin-1

**FIGURE 7:**
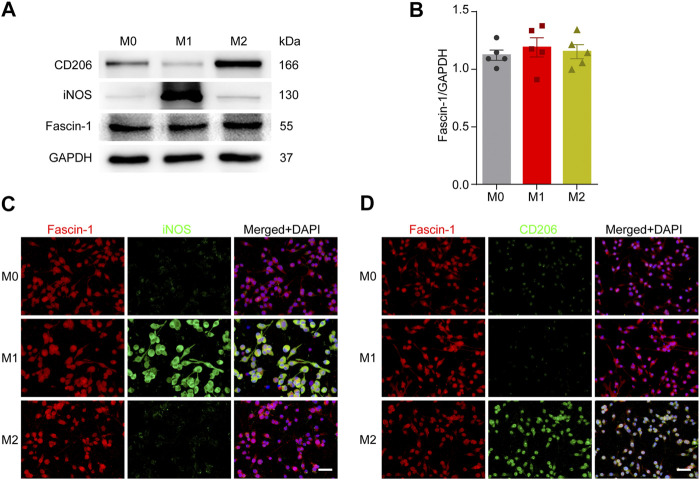
Microglial polarization has no effect on Fascin-1 expression. **(A)** Western blot analysis shows the protein expression of M1-like (iNOS) or M2-like (CD206) polarization markers and Fascin-1 in BV-2 cells after polarization treatment. **(B)** Quantitative analysis of Fascin-1 expression in **(A)**. GAPDH was used as the loading control. The blots (*n* = 5 per group) were quantified, as previously described, and no significant difference was observed. **(C,D)** Representative immunofluorescence images of M1-like (iNOS, Green) or M2-like (CD206, Green) markers and Fascin-1 (Red) in BV-2 cells after polarization treatment. The results also indicated no obvious differential expression of Fascin-1. DAPI (Blue) was used to stain the nuclei. Scale bar: 50 μm.

Previous studies have shown that microglia can be polarized into either a pro-inflammatory (M1-like) or anti-inflammatory (M2-like) phenotype and involved in the regulation of the SCI microenvironment (Francos‐Quijorna et al., 2016). Next, we detected the effect of microglial polarization on Fascin-1 expression. Western blot analysis showed that M1-like microglia significantly expressed iNOS and M2-like microglia significantly expressed CD206 (Figure 7A). In addition, immunocytochemistry was used to further confirm the reliability of microglial polarization (Figures 7C,D). However, the expression of Fascin-1 in the polarized microglial populations was not significantly different (*p* > 0.05, Figure 7). The results show that microglial polarization has no effect on the expression of Fascin-1.

## Discussion

Microglia, the resident immune cells of the CNS, are cells that rapidly respond to CNS injury (Davalos et al., 2005; Nimmerjahn et al., 2005). Following SCI, these cells migrate or project processes toward sites of injury, where they release neurotrophic agents and confer neuroprotection (Bellver-Landete et al., 2019; Plemel et al., 2020). In this study, we demonstrate that the actin-bundling protein Fascin-1 is highly expressed specifically in microglia after SCI and is indispensable for microglial migration. The expression of Fascin-1 is correspondingly reduced after the specific elimination of microglia by the CSF1R inhibitor PLX5622, and this effect is associated with disordered astrocytic and fibrotic scars and widespread macrophages in the injured spinal cord. Thus, we highlight that Fascin-1 can play a vital role in regulating microglial migration and microglial scar formation after SCI.

The proliferation of CX3CR1^+^ residual microglia has been observed beginning at 3 days after SCI (Wang et al., 2015). Microglia are rapidly recruited around the lesion epicenter, exhibit an ameboid morphology, and upregulate the lysosome-associated protein CD68, which suggests a potential increase in their phagocytic activity. Moreover, microglia continue to proliferate and accumulate around the site of the lesion epicenter, peaking at 14 days after SCI and forming microglial scars (Bellver-Landete et al., 2019). Our results also show that Fascin-1 is prominently expressed in microglia beginning at 3 days and peaks at 14 days after SCI. Olah et al. determined the global gene expression changes in microglia during demyelination and remyelination via microarray analysis, and the results suggested that the primary functions of CNS-resident microglia are repair and maintenance of tissue homeostasis (Olah et al., 2012). Using the lysozyme M EGFP-knock-in mouse, in which the expression of EGFP is specifically promoted in hematogenous macrophages but in not microglia, Greenhalgh and David revealed that microglia are the predominant cells that contact and phagocytose tissue debris 3 days after SCI, but then this role is taken by infiltrating macrophages. Macrophages seem to be more efficient in phagocytosing CNS debris but less efficient in clearing debris. Because peripheral macrophages are much more susceptible to apoptotic and necrotic cell death than CNS microglia after phagocytosing debris, which may contribute to the secondary damage after SCI (Greenhalgh and David, 2014). In the latest study, the transplantation of CX3CR1^GFP/+^ neonatal microglia or adult microglia treated with peptidase inhibitors into the spinal cord lesions of adult mice improved wound healing and axon regeneration (Li et al., 2020). Consistent with previous studies, our results also confirmed that lesion-localized Mac2^+^ macrophages are surrounded by CX3CR1^+^ microglia outside of the lesion at 14 days after SCI. Moreover, when microglia are eliminated, the compact form of astrocytic scars is destroyed, and macrophages are scattered, indicating that microglial scarring plays an important role in maintaining astrocytic scarring and limiting inflammation. However, the mechanism by which microglia accumulate to form protective scars remains unclear.

Fascin-1 is a cytoskeleton-organizing protein localized at the core actin bundles within microvillar projections and filopodial extensions of migrating cells (Ishikawa et al., 2003; Hayashi et al., 2008). Fascin-1 is expressed in neurons, dendritic cells and myofibroblasts (Al-Alwan et al., 2001; Tan et al., 2013). Fascin-1 can promote structural changes in cell membranes and affect the integrity of intercellular interactions to promote the invasion and metastasis of tumor cells (Hashimoto et al., 2009; Kim et al., 2009). Compared with the vector control, overexpression of Fascin-1 in colonic epithelial cells increases their motility on two-dimensional laminin surfaces and enhances their migration through extracellular matrix (ECM)-coated filters (Hashimoto et al., 2005). However, tumor metastasis of lung adenocarcinoma cells is blocked when Fascin-1 is knocked out with short hairpin RNA (shRNA), indicating that Fascin-1 plays a mechanical role in driving tumor cell migration and invasion (Lin et al., 2019). These studies show that Fascin-1 may be essential for cell migration. In the CNS, Fascin-1 plays an indispensable role in the development and polarization of filopodia (early neuritis) and growth cones, which can guide neurite outgrowth and branching (Pak et al., 2008). Fascin-1 can also bind to MHC-II and B7-2 to play a role in the antigen presentation of dendritic cells (Al-Alwan et al., 2001). B Wang et al. studied the role of microglia in a sciatic nerve injury model of rat neuropathic pain and found that microglia upregulate the expression of Fascin-1 in the dorsal horn of the L4-L6 spinal cord, which then participates in the process of antigen presentation and the regulation of the secretion of the inflammatory factors TNF-α and IL-6 (Wang et al., 2018). Considering that microglia are professional antigen-presenting cells in the CNS, we hypothesized that Fascin-1 may also regulate the migration and functional activity of microglia after SCI. Our data show that Fascin-1 is highly expressed specifically in microglia after SCI but not in neurons, astrocytes, NG2^+^ cells, pericytes, or blood-derived macrophages. Hence, Fascin-1 may be a specific marker of the activated microglia during CNS injury. Fascin-1 also plays an important role in regulating the migration of microglia, as demonstrated by gain- and loss-of-function studies. The specific effect of Fascin-1 on inflammatory response and microglial scars formation should be further confirmed using microglial *Fascin-1-conditional knockout (CKO)* transgenic mice in which the Fascin-1 gene is deleted under the CX3CR1 promoter.

CX3CR1 is a receptor of fractalkine and directly mediates the adhesion and migration of leukocytes and microglia (White and Greaves, 2012; Chidambaram et al., 2020). Wang X et al. labeled infiltrating macrophages in chimeric mice and showed that after SCI, infiltrating macrophages expressing higher Mac2 levels accumulated at the epicenter and microglia expressing higher CX3CR1 levels were distributed at the edges of the lesion (Wang et al., 2015). In CX3CR1^−/−^ mice with demyelinating disease, the clearance of myelin debris by microglia was substantially inhibited, affecting the integrity of the axon and myelin sheaths and thus preventing remyelination (Lampron et al., 2015). Moreover, we found that Fascin-1 is expressed specifically in CX3CR1^+^ activated microglia after SCI, and the expression of Fascin-1 decreases accordingly with the depletion of CX3CR1^+^ microglia. We hypothesize that there may be a correlation between CX3CR1 and Fascin-1, which need to be further studied. Unfortunately, we have not elucidated the effect of Fascin-1 on functional recovery of mice after SCI. The construction of *Fascin-1-CKO* transgenic mice to clarify the effect of Fascin-1-mediated migration of microglia on the functional recovery after SCI requires further exploration.

Myelin debris increases within the first week after SCI and persist in the injured spinal cord during the demyelination process (Kopper and Gensel, 2018). Clearance of myelin debris by phagocytes is necessary for SCI repair. Some studies indicate that uptake of myelin skews microglia/macrophages towards an immunosuppressive and neurotrophic phenotype (Boven et al., 2006; Bogie et al., 2011), but sustained uptake and intracellular accumulation of myelin leads to the formation of foamy macrophages and skews these cells towards an pro-inflammatory and neurotoxic phenotype (Wang et al., 2015; Cantuti-Castelvetri et al., 2018; Bogie et al., 2020). Our study shows that myelin treatment can up-regulate the expression of Fascin-1 and promote the migration of microglia *in vitro*. This may be related to the formation of neuroprotective microglial scar. Microglia play an important role in inflammation and nerve remodeling (Hambardzumyan et al., 2016). It has been shown that microglia can be polarized into M1-neurotoxic or M2-neuroprotective states and produce a variety of cytokines involved in the regulation of the SCI microenvironment (Hu et al., 2015; Han et al., 2018). However, in this study, we found that the polarization of microglia does not affect the expression of Fascin-1. The shortcomings of this article are that it did not do functional verification *in vivo* and further explore the effect of Fascin-1 on the cellular immune phenotype. Moreover, whether there is an interaction between microglia polarization and Fascin-1 expression requires further study. Nevertheless, our current experimental results could be preliminarily drawn to the existing conclusions.

In summary, this study found that Fascin-1 is highly expressed specifically in microglia after SCI and can regulate microglial migration. The migration and accumulation of microglia at the lesion border after SCI may be closely related to the specific upregulation and cellular localization of Fascin-1. Hence, the elucidation of this mechanism will provide new insights into novel therapeutic targets for the treatment of SCI.

## Data Availability

The original contributions presented in the study are included in the article/Supplementary Material, further inquiries can be directed to the corresponding authors.
